# Isorhamnetin: what is the *in vitro* evidence for its antitumor potential and beyond?

**DOI:** 10.3389/fphar.2024.1309178

**Published:** 2024-04-08

**Authors:** Jiaming Lei, Jianbao Yang, Cuiyu Bao, Feifei Lu, Qing Wu, Zihan Wu, Hong Lv, Yanhong Zhou, Yifei Liu, Ni Zhu, You Yu, Zhipeng Zhang, Meichun Hu, Li Lin

**Affiliations:** ^1^ Key Laboratory of Environmental Related Diseases and One Health, School of Basic Medical Sciences, Xianning Medical College, Hubei University of Science and Technology, Xianning, China; ^2^ School of Public Health, Xianning Medical College, Hubei University of Science and Technology, Xianning, China; ^3^ Hubei Province Key Laboratory on Cardiovascular, Cerebrovascular and Metabolic Disorder, Xianning Medical College, Hubei University of Science and Technology, Xianning, China; ^4^ School of Biomedical Engineering, Xianning Medical College, Hubei University of Science and Technology, Xianning, China; ^5^ Department of Medical School of Facial Features, Xianning Medical College, Hubei University of Science and Technology, Xianning, China

**Keywords:** isorhamnetin (ISO), pharmacological activity, antitumor, organ protection, mechanism

## Abstract

Isorhamnetin (ISO) is a phenolic compound belonging to flavonoid family, showcasing important *in vitro* pharmacological activities such as antitumor, anti-inflammation, and organ protection. ISO is predominantly extracted from *Hippophae rhamnoides L*. This plant is well-known in China and abroad because of its “medicinal and food homologous” characteristics. As a noteworthy natural drug candidate, ISO has received considerable attention in recent years owing to its low cost, wide availability, high efficacy, low toxicity, and minimal side effects. To comprehensively elucidate the multiple biological functions of ISO, particularly its antitumor activities and other pharmacological potentials, a literature search was conducted using electronic databases including Web of Science, PubMed, Google Scholar, and Scopus. This review primarily focuses on ISO’s ethnopharmacology. By synthesizing the advancements made in existing research, it is found that the general effects of ISO involve a series of *in vitro* potentials, such as antitumor, protection of cardiovascular and cerebrovascular, anti-inflammation, antioxidant, and more. This review illustrates ISO’s antitumor and other pharmacological potentials, providing a theoretical basis for further research and new drug development of ISO.

## 1 Introduction

**FIGURE 1:**
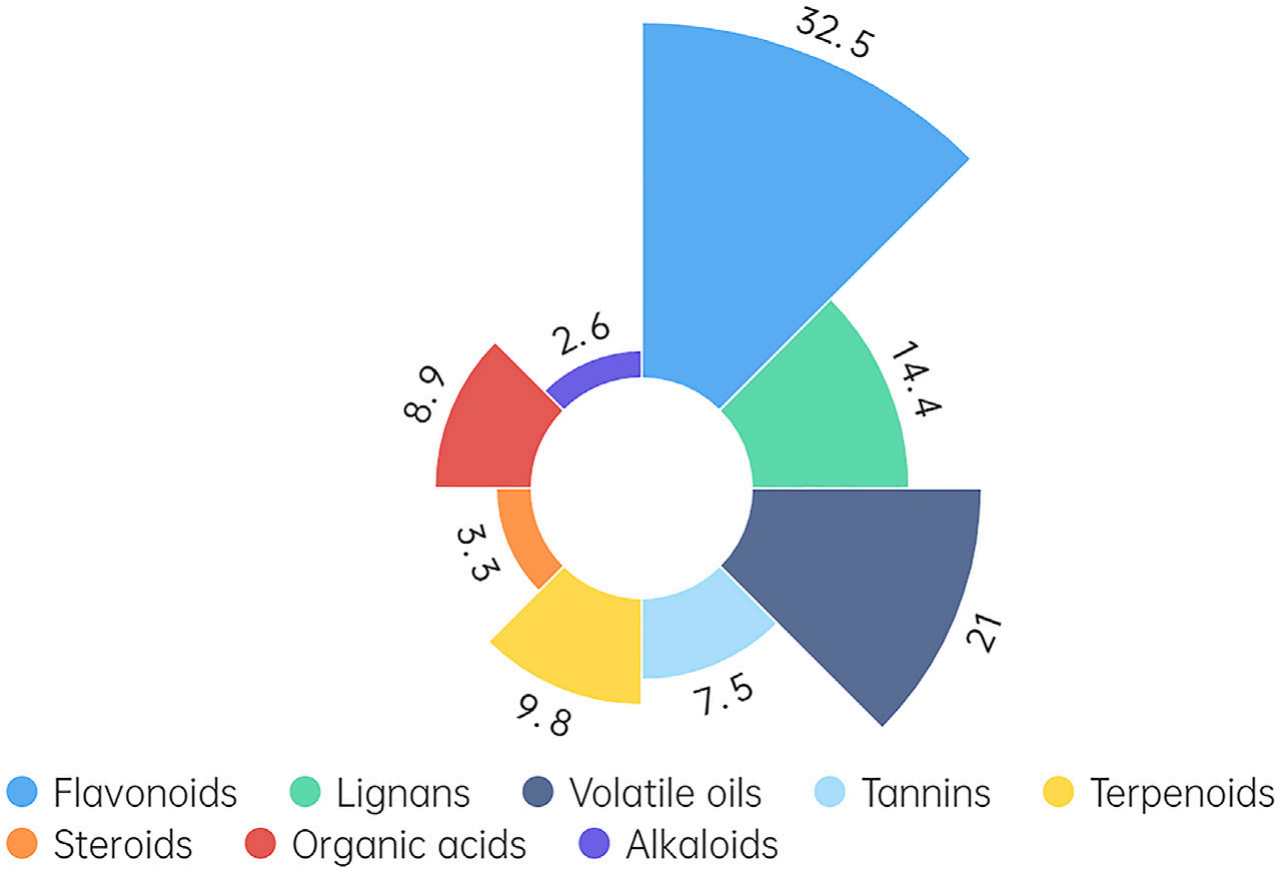
Types and magnitude ratios of compounds in *Hippophae rhamnoides L.*

Isorhamnetin (ISO), a flavonoid predominantly found in fruits and leaves of various plants, including *Hippophae rhamnoides L. (H. rhamnoides L.)*, is renowned for its diverse pharmacological effects (Gong et al., 2020). *H. rhamnoides L.* is a medicinal and food homologous plant reported to contain over 190 bioactive components (Ma et al., 2023). The plant, including its fruits, seeds, leaves, roots, and branches, is rich in nutrients and chemicals. Notably, it encompasses numerous pharmacologically active components characterized by diverse chemical structures (Han et al., 2021; Pirintsos et al., 2022). Eight functional components have been isolated from *H. rhamnoides L.* primarily composed of flavonoids (32.5%), lignans (14.4%), volatile oils (21.0%), tannins (7.5%), terpenes (9.8%), steroids (3.3%), organic acids (8.9%), alkaloids (2.6%) (Figure 1) (Ma et al., 2021; Feng et al., 2023). Except for *H. rhamnoides L.*, ISO can also be detected in Ginkgo biloba (*Ginkgo biloba L.*). Standard extract of *G. biloba L.* includes almost 6% terpene lactones (2.8%–3.4% ginkgolides A, B, and C and 2.6%–3.2% bilobalide) and 24% flavone glycosides (isorhamnetin, quercetin, and kaempferol) (Eisvand et al., 2020). The content of ISO in *H. rhamnoides L.* is obviously higher than that in *G. biloba L.* Currently, researches on ISO mainly focus on exploring its extraction and medicinal properties in *H. rhamnoides L.*

Natural small molecule compounds, characterized by their diversity, accessibility, and structural controllability, have emerged as promising alternatives. They effectively induce cell differentiation with minimal adverse effects (Almatroodi et al., 2021; Lu et al., 2023), presenting therapeutic potential for various severe diseases, including highly malignant and drug-resistant cancers (Ouyang et al., 2014; Tan et al., 2023). Cancer stands as one of a leading cause of mortality with increasing incidence year by year (Siegel et al., 2023; Sung et al., 2021). Contemporary cancer treatments involve surgery, postoperative radiotherapy, and chemotherapy, yet their prolonged use is hindered by high toxicity and side effects, resistance to classical anti-cancer drugs, and high cost (Zughaibi et al., 2021; Lattard et al., 2023). Flavonoids, including ISO, have garnered attention for their varied biological properties, such as inhibition of cell proliferation, angiogenesis, multidrug resistance reversal, and the therapeutic potential of various cancers (Ul Islam et al., 2021). In addition, there are a variety of other pharmacological activities. Therefore, ISO has gradually become a research hotspot in recent years (Sun et al., 2022). Besides cancers, cardiovascular and cerebrovascular protection, prevention of obesity, anti-inflammatory, antioxidant and other effects also capture the focus of research field in phytomedicine (Lv et al., 2023).

ISO, categorized under flavonoids, represents a natural single compound isolated from total flavonoids (Lv et al., 2023). The pharmacological activity of ISO has been extensively discussed in previous research (Gong et al., 2020), however, a systematic exploration is lacking. This review primarily focuses on the following 5 aspects: 1) The overarching traits of ISO encompass ethnopharmacology and physicochemical characteristics; 2) ISO’s potential antitumor effects across different cancers; 3) The mechanism of ISO’s combined therapy for cancer; 4) The organ protective effect and mechanism of ISO; 5) Other pharmacological activities of ISO. The review aims to highlight the application prospects of ISO as a potential therapeutic drug.

## 2 General characteristics of ISO

### 2.1 Ethnopharmacology

**FIGURE 2:**
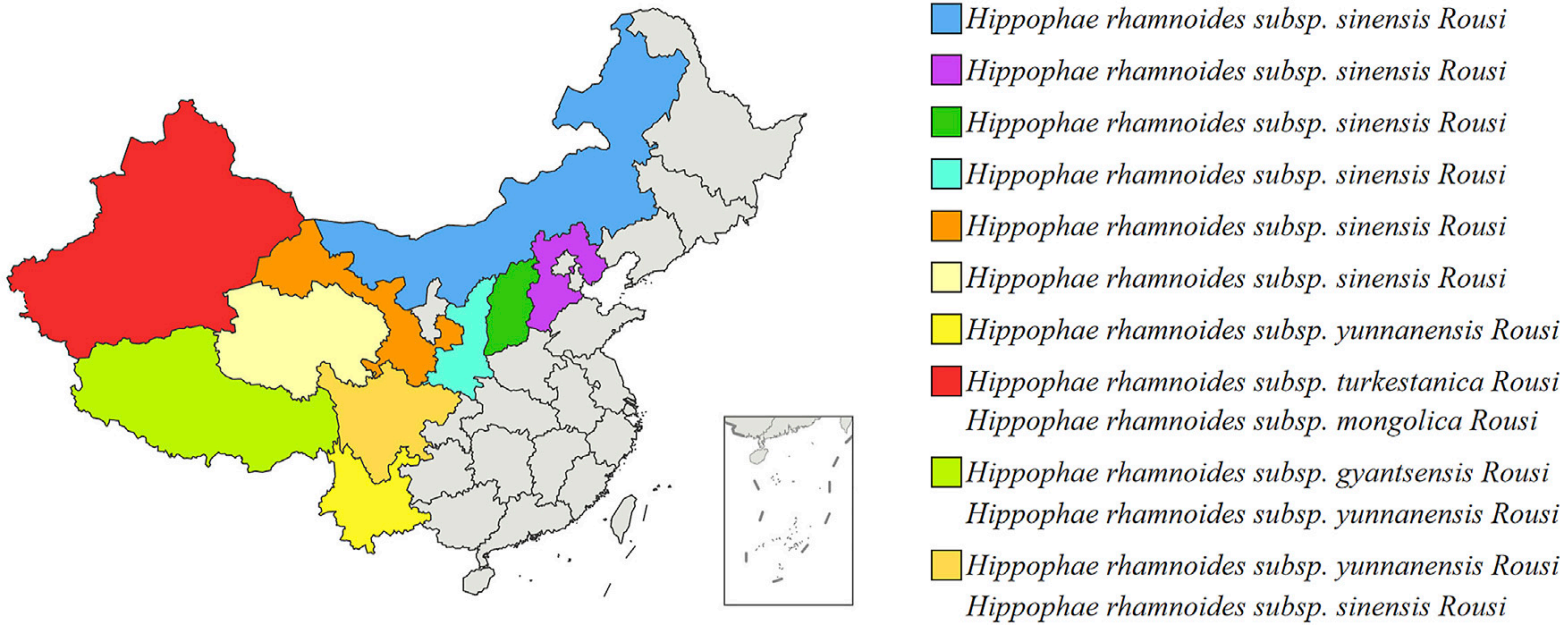
Distribution of various provinces of China *Hippophae rhamnoides L.*

*H. rhamnoides L.* is indigenous to Eurasia and is mainly distributed in China, Greece, Mongolia, Tajikistan, Afghanistan, Russia, Turkey, and India. The characteristics of the *H. rhamnoides L.* are described detailly in Table 1 (Ma et al., 2023). There are 5 subtypes of *H. rhamnoides L.*, which includes *H. rhamnoides subsp. Mongolica Rousi, H. rhamnoides subsp. Gyantsensis Rousi, H. rhamnoides subsp. yunnanensis Rousi, H. rhamnoides subsp. Sinensis Rousi,* and *H. rhamnoides subsp. Turkestanica Rousi.* It is mainly distributed in North China, Northwest China and Southwest China. Figure 2 shows the distribution of *H. rhamnoides L.* in China (Ciesarová et al., 2020).

**TABLE 1:** The characteristics and distribution of *Hippophae rhamnoides L.* in China.

Name	Distribution	Characteristic	References
*Hippophae rhamnoides subsp. mongolica* *Rousi*	Xinjiang	Plant: 2–6 m tall; Young shoots grey or brown, old branches thick, lateral spines long and slender, often unbranched	www.iplant.cn
		Leaves: leaves alternate, 40–60 mm long, 5–8 mm wide, widest above middle, blunt apex, green or slightly silvery white above	
		Fruit: fruit round or nearly round, 6–9 mm long, 5–8 mm diameter, peduncle 1–3.5 mm long	
*Hippophae rhamnoides subsp.gyantsensis Rousi*	Tibet	Plant: 5–8 m tall, small branches slender, gray or brown; The internodes are shorter	www.iplant.cn
		Leaves: leaves alternate, papery, narrow-lanceolate, 30–55 mm long, 3–5 mm wide, broadest at base, blunt apex, margins fully margined, slightly reversed, green or slightly whitish above, with scattered star-like white pubescent or villi, especially midrib, greyish-white below, densely covered with silvery-white and scattered few brown scales, sometimes scattered white villi, midrib sunken above, conspicuously convex below; The petioles are extremely short or almost absent	
		Fruit: Fruit oval, 5–7 mm long, 3–4 mm diameter, yellow; peduncles about 1 mm long	
*Hippophae rhamnoides subsp. yunnanensis* *Rousi*	Sichuan, Yunnan, Tibet	Leaves: leaves alternate, broadest at base, often rounded or sometimes wedge-shaped, covered with rust-colored scales below	www.iplant.cn
		Fruit: fruit spherical, 5–7 mm diameter, peduncle 1–2 mm long	
		Seeds: broadly elliptic, slightly flattened, 3–4 mm long	
*Hippophae rhamnoides subsp. sinensis* *Rousi*	Nei Mongol, Hebei, Shanxi, Gansu, Shaanxi, Qinghai, Sichuan	Plant: up to 5 (−18) m	www.iplant.cn
		Leaves: leaves nearly opposite, papery, lanceolate or oblong-lanceolate, 3–8 cm long, 0.4-1 (−1.3) cm wide, blunt at both ends or nearly rounded at base, green above, initially covered with white shield-shaped or stellate pubescence; silvery-white below, scaly, without stellate pubescence; petioles absent or 1–1.5 mm long	
		Fruit: spherical, 4–6 mm in diameter, orange-yellow or orange-red when ripe; peduncles 1–2.5 mm long	
		Seeds: Seeds broadly oval or ovate, sometimes slightly flattened, 3–4.2 mm long, black or purplish-black, shiny	
*Hippophae rhamnoides subsp. turkestanica* *Rousi*	Xinjiang	Leaves: alternate, 1.5–4.5 cm long, 2–4 mm wide, silvery-white on both sides	www.iplant.cn
		Fruit: 5-7 (−9) mm long, 3–4 mm in diameter, brittle flesh when dry, peduncle 3–4 mm long	

According to historical records, *H. rhamnoides L.* is extensively employed for treating medical conditions in numerous countries. The *H. rhamnoides L.* is called “longevity fruit” by Japan, “second ginseng” by Russia, “life energy” by the United States, “divine fruit” by India, “sacred fruit” and “king of vitamin C” by China. *H. rhamnoides L.* a plant extensively utilized in both the Chinese Pharmacopoeia and the World Pharmacopoeia, is employed for treating various health-related issues, such as cough, skin diseases, jaundice, asthma, hypertension, rheumatism and genital inflammation traditionally. Traditional use of various plant parts of *H. rhamnoides L.* in various region has been summarized in Table 2 (Yang et al., 2001).

**TABLE 2:** Traditional uses of different parts of *Hippophae rhamnoides L.*

Geographical location	Plant part used	Disease and method of use	References
Tibet, China	seeds, fruits, leaves	edema, tissues regeneration, skin grafts, inflammations, bacterial infections, burns/injury caused by cosmetic laser surgery, corneal wounds	Yogendra Kumar et al. (2011)
Russia	berries	edema, tissue regeneration, skin graft, inflammation, bacterial infection, corneal wound	Suryakumar and Gupta (2011)
Sichuan, China	fruits, seed, leaves	spleen digestion, blood stasis, radiation damage, inflammation, burn, tissue regeneration, skin graft	Suryakumar and Gupta (2011)
Mongolia	leaves, branches	colitis, enterocolitis	Suryakumar and Gupta (2011)
India	fruits	digestive disease	Suryakumar and Gupta (2011)

Researchers assessed the total flavonoids and total polyphenols in *H. rhamnoides L.* from six origins using the Al (NO_2_)_3_-NaNO_2_-NaOH method and the Folin-phenol method, respectively. Furthermore, they assessed the antioxidant activity of *H. rhamnoides L.* extract by determining the scavenging rates of DPPH free radicals and ABTS free radicals (Uwineza and Waśkiewicz, 2020). The results show that there are some differences in the contents of total flavonoids and total polyphenols in *H. rhamnoides L.* from different origins. Among them, the contents of total flavonoids and total polyphenols in *H. rhamnoides L.* from Inner Mongolia are the highest, reaching 38.84 mg/g and 33.31 mg/g, respectively, while those from Gansu are the lowest, reaching 8.50 mg/g and 12.36 mg/g, respectively. The *H. rhamnoides L.* extracts from different origins have higher antioxidant activity, which have a certain scavenging effect on three kinds of free radicals, and there is a dose-effect relationship within a certain concentration range (Li et al., 2021). Interestingly, the content of flavonoids in *H. rhamnoides L.* from different habitats is different. Therefore, the extracted ISO also showed corresponding differences. ISO’s potential anti-tumor and other pharmacological activities will also show corresponding differences (Sasidharan et al., 2011).

### 2.2 Physical and chemical properties

**FIGURE 3:**
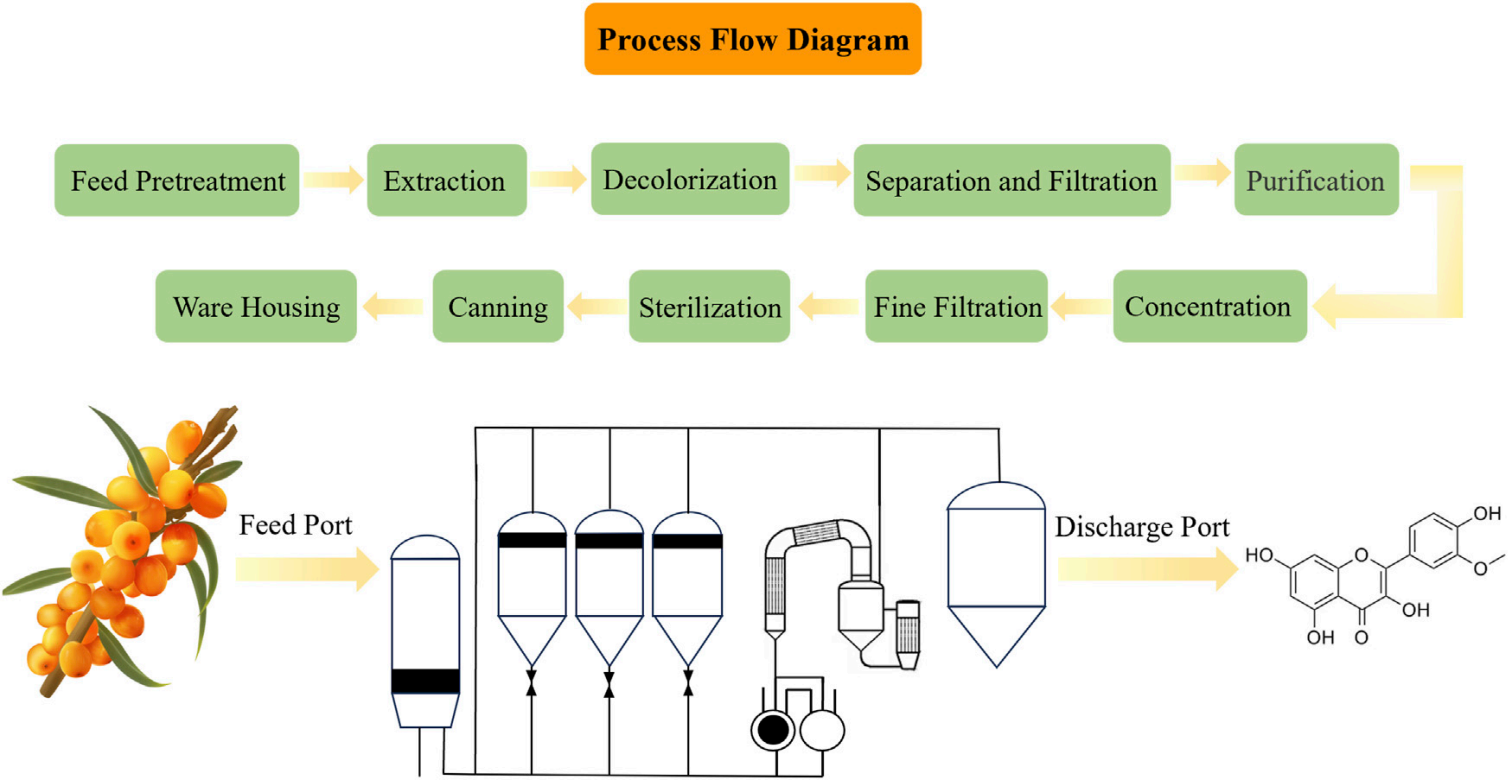
The phytoextraction of ISO from *Hippophae rhamnoides L.* Above diagram represents the process flow of phytoextraction of ISO. Left side below is a hand-drawing picture of *Hippophae rhamnoides L.*; the green is the leaf and the orange is the fruit of *Hippophae rhamnoides L.*, and then subjected to extract ISO. Middle below depicts the workflow of ISO extraction. The medicinal plant *Hippophae rhamnoides L.* are throwed into the feed port and the extracted ISO is obtained from the discharge port. Right side below is the chemical structure of ISO.

ISO is a small molecule compound characterized by an aromatic heterocyclic structure. (C_16_H_12_O_7_, CAS No. 480-19-3), classified under plant flavonoid (Li et al., 2022), with a chemical molecular weight of 316.26 Da. Its chemical structure is shown in Figure 3, consisting of two phenolic benzene rings, representing the flavonoid parent structure, alongside adjacent hydroxyl and methoxy groups. In addition, Figure 3 also shows the detailed ISO extraction process. There are also many methods for extracting flavonoids from *H. rhamnoides L.*, including solvent extraction, microwave-assisted extraction, soxhlet’s extraction, ordinary ultrasonic extraction and ultrasonic cycle extraction. Table 3 compares and analyzes the advantages and disadvantages of the above five different extraction methods. The ISO extracted by different extraction methods is different. Correspondingly, ISO’s potential anti-tumor and other pharmacological activities will show corresponding differences. Pure ISO appears as yellowish acicular crystal, demonstrating slight solubility in water and methanol, yet solubility in a mixed solvent of methanol and chloroform. ISO is noted for its diverse biological activities, with a particular emphasis on anti-oxidant activity, effectively scavenging free radicals and shielding cells from harmful substances (de Souza Farias et al., 2021). Investigated for applications in cardiovascular disease, anti-inflammatory, anti-oxidant, and anti-osteoporosis effects (Sager, 2023), ISO serves as a potential anti-cancer agent combating various cancer types, including breast cancer, lung cancer, prostate cancer, and others.

**TABLE 3:** Advantages and disadvantages of the extraction process of flavonoids from *Hippophae rhamnoides L.*

Extraction process	Advantages	Disadvantage	References
Solvent extraction	Low cost, simple operation, suitable for industrial production	The cost is high, and the organic solvent is easy to remain harmful to the human body	Zhou et al. (2020)
Microwave-assisted extraction	Shorten the extraction time and improve the extraction efficiency	Limited to the extraction of thermally stable substances	Zhou et al. (2022)
Soxhlet’s extraction	The operation is simple and easy, saving time	Easy to cause environmental pollution	Zhou et al. (2020)
Ordinary ultrasonic extraction	High efficiency, energy saving, environmental protection	Low extraction efficiency	Zhou et al. (2020)
Ultrasonic cycle extraction	Greatly improve the extraction rate	Easy to be restricted by ultrasonic attenuation	Zhou et al. (2020)

## 3 Anti-tumor effects of ISO *in vitro*

Cancer stands as a prominent global cause of mortality, with an annual death surpassing 10 million. Conventional cancer treatments encompass surgical intervention, radiation, and chemotherapy; however, these approaches often compromise healthy cells, causing toxicity in patients. Despite extensive efforts to enhance diagnostic techniques and therapeutic interventions, the overall prognosis for cancer patients remains notably grim. The quest for alternative preventive strategies and safe, efficient agents for cancer treatment is imperative (Desai et al., 2020). Traditional Chinese medicine, a natural treasure trove, presents an opportunity to discover novel compounds for cancer prevention (Crunkhorn, 2022). In recent years, ISO has emerged as effective in inhibiting the growth and development of various common malignancies (Table 4). In fact, ISO plays an important role in chemoprevention by activating major cellular defense mechanisms to combat metabolism, foreign bodies, and oxidative stress (Dong et al., 2022). ISO’s anti-cancer role involves cell cycle blockade, regulation of apoptosis and autophagy, and inhibition of cancer cell invasion and metastasis *in vitro*.

**TABLE 4:** Antitumor effects *in vitro* of ISO.

Tumor type	Target	Experimental model	Experimental dose	Control	References
Breast Cancer	AKT-MEK	MCF7, MDA-MB-48	0–30 μM 24 h	JSH-23	Hu et al. (2015)
Prostate Cancer	PI3K/AKT/mTOR	DU145, PC3	0–20 μM 24 h	Deguelin	Cai et al. (2020)
Lung Cancer	AKT/ERK1/2	A549	0–10 μM 48 h	N.S.	Luo et al. (2019)
Colon Cancer	PI3K-AKT-mTOR	SW480, HT29	0–40 μM 24 h	N.S.	Li et al. (2014)
Gastric Cancer	NF-κB	SNU-5, SNU-6, NUGC3, AZ521, AGS, MNK45	0–50 μM 24 h	N.S.	Manu et al. (2015)
		athymic nu/nu female mice	1 mg/kg		
Pancreatic Cancer	Ras/MAPK	PANG-1	0–80 μM 24 h	N.S.	Wang et al. (2018)
Gallbladder Cancer	AKT/PI3K	NOZ, GBC-SD	0–80 μM 24 h	N.S.	Zhai et al. (2021)
		BALB/c nude female mice	1 mg/kg		
Bladder Cancer	AMPK	T24, 5637	0–100 μM 24 h	SC79	Park et al. (2019)
Skin Cancer	MEK1 and PI3K	A431	0–40 μM 24 h	N.S.	Kim et al. (2011)
		athymic nude female mice	0–5 mg/kg		

### 3.1 Breast cancer

Breast cancer is a common malignancy in women in developing and developed countries (Liang et al., 2020; Zhang et al., 2022), exhibiting metastasis as a key malignant feature and a leading cause of death in affected individuals. Matrix metalloproteinases (MMPs) actively contribute to cancer cells through excessive extracellular matrix (ECM) degradation (Bader et al., 2020). Previous study demonstrated (Li et al., 2015) that ISO significantly inhibits breast cancer MDA-MB-231 cell invasion by downregulating MMP-2 and MMP-9 expression and activity. This inhibition is potentially associated with the suppression of p38/mitogen-activated protein kinase (MAPK) and STAT3. Therefore, the findings provide new evidence for the anti-cancer activity of ISO. However, the toxicity and dosage of the drug require validation through additional *in vivo* experiments.

In other studies, researchers also investigated the potential effects of ISO on breast cancer and examined the effects of ISO on the AKT/mammalian target of rapamycin (mTOR) and MAPK/MEK signaling cascades, which are two important signaling pathways for endocrine therapy resistance in breast cancer. The results of these studies demonstrate that the antiproliferative and pro-apoptotic effects of ISO in breast cancer are mediated via inhibition of the AKT/mTOR and MEK/ERK signaling pathways and provide a basis for pursuing the therapeutic significance and chemopreventive capabilities of ISO in breast cancer.

### 3.2 Prostate cancer

Prostate cancer, ranking as the second leading cause of cancer-related mortality in men (Xie et al., 2022a), experiences inhibition of proliferation and metastasis through ISO treatment. ISO significantly retards cell growth and induces lactate dehydrogenase (LDH) release in androgen-independent DU145 and PC3 prostate cancer cells, while it exhibits no impact on androgen-dependent LNCaP cells. Elevated LDH, associated with a dismal prognosis in many solid tumors (Zhang et al., 2015), is mitigated by ISO’s reported selective inhibition of the PI3K-AKT-mTOR pathway. Furthermore, ISO’s *in vitro* antitumor activity is attributed of apoptosis induction and the blockade of migration and invasion. These effects may be mediated through the induction of a mitochondrion-dependent intrinsic apoptotic pathway and inhibiting epithelial-mesenchymal transformation (EMT) and the PI3K/AKT/mTOR signaling pathway. In summary, these *in vitro* findings may propose ISO as a promising therapeutic candidate for treating androgen-independent prostate cancer (Cai et al., 2020).

### 3.3 Lung cancer

Lung cancer, ranking as the most prevalent cancer and the leading cause of cancer-related mortality globally (Zhou et al., 2020; Zhang et al., 2022), confronts inhibition of A549 cell growth and metastasis through ISO treatment. Research data suggests that ISO effectively hinders the growth of A549 cell growth in a dose-dependent manner and impedes cell metastasis by reducing the activity of MMP-2/MMP-9 in A549 cells. In addition, EMT promotes the invasion and migration of solid tumor cells and is considered an important target for anticancer drug treatment (Chanvorachote et al., 2022). ISO interferes with the EMT process by inhibiting the PI3K/AKT/ERK signaling pathway to inhibit the metastasis of A549 cells (Ruan et al., 2015). Consequently, all these *in vitro* evidences verify ISO emerges as a potential antitumorigenic compound for lung cancer treatment and/or prevention in the future.

### 3.4 Colon cancer

Colon cancer, constituting a malignant tumor within the digestive tract system, has ascended to become the third most common tumor in China and the fourth leading cause of cancer-related death worldwide (Jaramillo et al., 2010; Peng et al., 2020). Research data suggest that ISO induces cell cycle arrest in the G2/M phase in a dose-dependent manner, thereby inhibiting the proliferation of colon cancer HT-29, HCT116, and SW480 cells. Mechanically, ISO inhibits cell proliferation by inhibiting the PI3K-AKT-mTOR pathway, reducing its phosphorylation level, and enhancing the expression of cyclin. The PI3K-AKT-mTOR signaling pathway, known for its pivotal role throughout the cell life cycle and viability as a target for anti-cancer drugs (Yap et al., 2008), signifies ISO’s selective inhibition on this pathway as an effective mechanism for treating colon cancer (Li et al., 2014).

### 3.5 Gastric cancer

Gastric cancer is a fatal malignancy and the second most prevalent cause of cancer-related death. While advancements in treatment options have contributed to a decline in gastric cancer-related fatalities, chemical resistance remains a pivotal factor in prognostic outcomes and the recurrence of treatment failure (Ramachandran et al., 2012). ISO treatment has demonstrated a profound inhibition effect on proliferation of two gastric cancer cell lines (AGS-1 and HGC-27). Notably, ISO-induced mitochondrial dysfunction was observed through the assessment of mitochondrial membrane potential (MMP), implicating its role in promoting apoptosis (Sighel et al., 2021). Further investigations unveiled that ISO triggerred apoptosis in gastric cancer cells through the mitochondria-dependent apoptotic pathway. Further study shows that ISO treatment in gastric cancer cells initiated the activation of the Caspase-3 cascade, including the upregulation of cytochrome C, Bax/Bcl-2, and the cleavage of Caspase-3 as well as PARP, and finally resulted in mitochondrial homeostasis imbalance and apoptosis. Therefore, these results indicate that ISO treatment induces the apoptosis of gastric cancer cells through the mitochondria-dependent apoptotic pathway, providing a potential strategy for clinical gastric cancer therapy.

### 3.6 Pancreatic cancer

Pancreatic cancer, notorious for its malignant nature and poor prognosis due to late detection, emerges as a formidable disease with a high risk of mortality (Pijnappel et al., 2022). ISO has exhibited significant inhibitory effects on the growth of PANC-1 pancreatic cancer cells, primarily by impeding the cell cycle S phase through inhibiting the activity of the Ras/MAPK pathway. These findings suggest that ISO holds promise as a novel prophylactic agent in chemotherapy prophylaxis for pancreatic cancer (Wang et al., 2018).

### 3.7 Gallbladder cancer

Gallbladder cancer, the most prevalent biliary tract tumor with a bleak prognosis, relies on radical surgery for effective early-stage cure (Berger et al., 2022). Recognizing the critical role of the PI3K/AKT in cell growth and survival, and it is one of the most promising avenues for the development of anti-gallbladder cancer drugs (Porta et al., 2014). Studies have shown that ISO effectively inhibits the proliferation and migration of gallbladder cancer NOZ and GBC-SD cells in a time- and dose-dependent manner. ISO’s pharmacological activity on gallbladder cancer was also assessed employing a mice xenograft model and immunohistochemistry staining. ISO was found to suppress cell proliferation and metastasis, trigger apoptosis and arrest the G2/M phase in gallbladder cancer cells via the inactivation of the PI3K/AKT signaling cascade. These findings provide new approaches and insights for the treatment of gallbladder cancer. Future research should prioritize the metabolic distribution of drugs in the body.

### 3.8 Bladder cancer

Bladder cancer is among the most prevalent high-incidence tumors and demonstrates a higher recurrence rate than other cancers, contributing to elevated mortality. Study have shown that ISO induces G2/M phase cell arrest and apoptosis. In addition, ISO decreases the expression of Wee1 and cyclin B1 but increases the expression of cyclin-dependent kinase (Cdk) inhibitor p21WAF1/CIP1, and increased p21 is bound to Cdk1. ISO’s inhibition on bladder cancer was also reported to be closely associated with cancer treatment by inducing apoptosis and regulating the cell cycle (Dillon et al., 2014). In addition, ISO enhances mitochondrial dysfunction, reflected in an elevated Bax/Bcl-2 expression ratio and cytochrome C release into the cytoplasm. Moreover, the induction of G2/M arrest and apoptosis by ISO was accompanied by activation of the AMPK signaling pathway, and excessive production of ROS. However, artificial interception of the AMPK signaling pathway attenuated ISO-induced apoptosis, and the interruption of ROS generation led cells to escape from G2/M arrest and apoptosis. These findings suggest that ISO holds chemopreventive potential by inducing G2/M arrest and apoptosis through ROS-dependent activation of the AMPK signaling pathway in bladder cancer cells.

### 3.9 Skin cancer

Skin cancer, a prevalent malignancy affecting millions globally, experiences an annual increase in incidence (Rojas et al., 2022). Reports reveal that the anti-skin cancer effects of ISO are mediated through inhibiting the epidermal growth factor (EGF) induced neoplastic cell transformation. ISO was also reported to suppress anchorage-dependent and -independent growth of A431 human epithelial carcinoma skin cancer cells. Besides, ISO attenuated EGF-induced COX-2 expression in skin cancer JB6 and A431 cells. In a mouse xenograft using skin cancer A431 cells, ISO reduced tumor growth and COX-2 expression. Among them, COX-2 was overexpressed in various tumors and played an important role in skin cancer (Jin, 2023). These results suggest that COX-2 is a target of ISO, exerting anticancer activity through the MEK and PI3K signaling pathways in the treatment of skin cancer.

**FIGURE 4:**
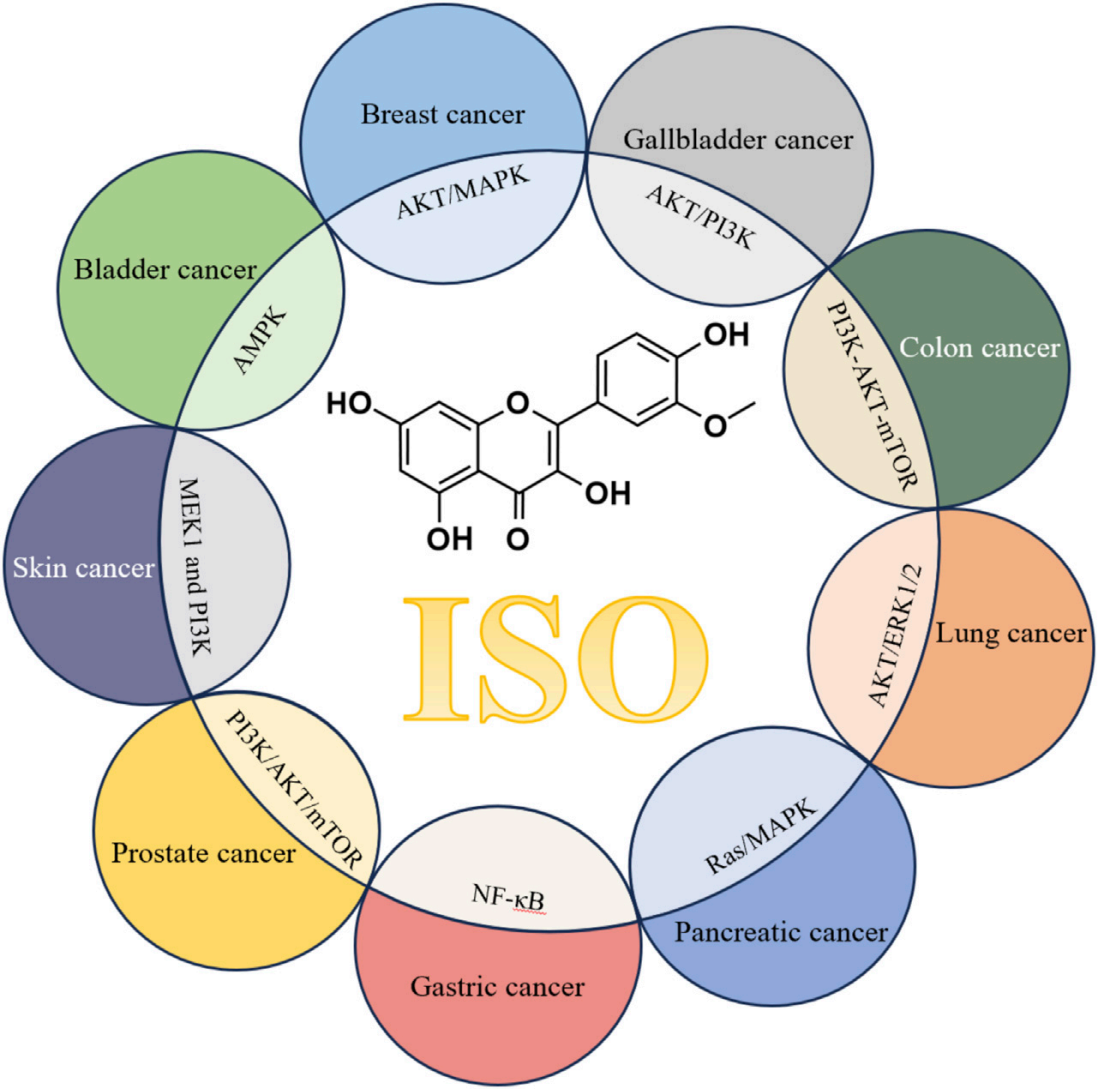
Schematic mechanism of ISO’s anti-tumor activity *in vitro* and involved molecular targets.

Figure 4 summarizes the *in vitro* anti-tumor activity and mechanism of ISO, which suggests that ISO may exert its pharmacological efficacy in combating cancer through various oncoprotein targets. While these studies have indicated that ISO may have anti-tumor effects, further investigations are necessary to thoroughly assess its efficacy and safety as an anti-tumor drug for future clinical applications. Carefully designed clinical trials must be conducted to select the appropriate patient, administration and measurement results to determine the potential of ISO as an anti-tumor drug.

## 4 ISO’s combined treatment of anti-tumor effects *in vitro*

Combination therapy refers to the simultaneous or sequential application of two or more drugs to achieve therapeutic purposes (Hu et al., 2024). Combination therapy’s objection is mainly to increase the efficacy or to reduce the toxic and side effects of a single drug (Nadova et al., 2007). Meanwhile, it can also delay or reduce the occurrence of drug resistance in addition to the synergistic effect of drugs to improve the efficacy, and reduce the dose of individual drugs (Yang et al., 2023).

Recent studies have reported that ROS-mediated activation and mitochondrial translocation of CaMKII contribute to Drp1-dependent mitochondrial fission and apoptosis in triple-negative breast cancer (TNBC) cells by ISO and chloroquine (CQ) (Hu et al., 2019). The impact of combined treatment with chloroquine (CQ) and ISO on cell viability were investigated in various human breast cancer cell lines, MDA-MB-231 and BT549. The synergistic effects of CQ/ISO on apoptosis were examined in MDA-MB-231 and BT549 cells. The combination of minimally toxic concentrations of CQ (20 μM) and ISO (10 μM) resulted in a pronounced increase in apoptosis in MDA-MB-231 and BT549 cells. Additionally, the impact of CQ on the inhibitory efficacy of ISO using a TNBC xenograft mouse model. Following inoculation, mice received injections of either vehicle, CQ (40 mg/kg), ISO (20 mg/kg) or a combination of these for 80 days (n = 10), and these findings indicate that the inhibition of autophagy by CQ can be sensitized to ISO -induced cell death.

The emergence of drug resistance to standard chemotherapy is a common phenomenon that often leads to a poor prognosis. A recent study has shown that ISO enhances the anti-tumor effect of capecitabine through the negative regulation of the NF-κB signaling cascade in gastric cancer (Manu et al., 2015). ISO significantly inhibited the growth of two multidrug-resistant human gastric cancer cells (NUGC3 and AZ521) in a dose- and time-dependent manner. Results of flow cytometric analysis clearly indicated that the sub-optimal doses of ISO (10 μM) or capecitabine (10 μM) had a minimal effect on apoptosis alone but caused a substantial increase in programmed cell death when used in conjunction of these two drugs. The anti-tumor efficacy of ISO and capecitabine alone and/or in combination with each other was further investigated in a gastric cancer xenograft mouse model. The combination treatment group exhibited a significant reduction in tumor volume compared to the control group or the capecitabine-alone group (60 mg/kg, oral gavage twice a week).

It is worth noting that, in the context of safety and addressing drug resistance, combination therapy is regarded as a promising treatment option, and further efforts are needed to improve well-designed clinical trials and the clinical potential of ISO.

## 5 *In vitro* organ protection effects of ISO

The *in vitro* antitumor activities of ISO are described in detail above (Table 4), while the potential *in vitro* organ protective effects of ISO are reviewed in the following sections. Organ diseases, such as traumatic brain injury, ischemic stroke, myocardial ischemia-reperfusion injury, acute lung injury, acute kidney injury, etc. remain inadequately treated, significantly impacting the prognosis of patients. Various studies have proved that ISO has a wide range of organ protective effects, including the cardiovascular system, nervous system, liver, and other important organs. The protective mechanism of ISO on organ damage opens new avenues for the treatment of organ diseases.

### 5.1 Cardiovascular protection

Cardiovascular disease (CVD) is the leading cause of death in Western countries, accounting for nearly 30% of global fatalities. In recent years, CVD exhibited an alarming trend of affecting individuals at younger ages, posing an escalating threat to human health. Studies have reported that ISO has biological activity in the cardiovascular system (Li et al., 2022). As one of the inflammatory cytokines, tumor necrosis factor-alpha (TNF-α) is an important risk factor in the process of arteriosclerosis (Bae et al., 2022; Xie et al., 2022b). Endothelial nitric oxide synthase (eNOS) plays an important role in the regulation of cardiovascular function. In general, eNOS is expressed in responses to inflammatory stimuli such as cytokines and generates large quantities of nitric oxide (NO) (Ponnuswamy et al., 2012), which acutely vasodilates blood vessels and inhibits platelet aggregation. Generally, eNOS assumes a pivotal role in the regulation of prevention of atherosclerosis by decreasing leukocyte adhesion and smooth muscle proliferation (Förstermann and Münzel, 2006). The protective effects of ISO on apoptosis and inflammation in TNF-α-induced HUVECs injury were studied, and results showed ISO inhibited TNF-α-induced apoptosis and upregulation adhesion molecules ICAM-1, VCAM-1, E-selectin by suppressing the NF-κB and AP-1 expression. Simultaneously, up regulating the expression of eNOS by ISO was involved in the protective process which could help to keep vascular function. These findings suggest that ISO has the potential effects of anti-apoptosis and anti-inflammation on TNF-α induced HUVECs, offering a plausible underlying mechanism for the treatment of coronary heart disease (Nebigil and Désaubry, 2018).

### 5.2 Hepatoprotective effects

Hepatic fibrosis is a critical factor in the progression of chronic liver diseases, ultimately leading to the development of cirrhosis and hepatocellular carcinoma (Machicado et al., 2016). Activation of hepatic stellate cells (HSCs) stands as the primary cause of hepatic fibrogenesis. A previously published study revealed that by investigating the ability of ISO to protect against hepatic fibrosis *in vitro*, ISO inhibited TGF-β1-induced expression of α-smooth muscle actin (α-SMA), plasminogen activator inhibitor-1 (PAI-1), and collagen in primary murine HSCs and LX-2 cells. TGF-β1 has been characterized as a key mediator in the process of liver fibrosis (Li and Wu, 2020). The effect of ISO on HSCs activation occurs through a canonical TGF-β signaling pathway. In addition, oxidative stress is the main cause of liver fibrosis (Forman and Zhang, 2021). Some published literatures showed that ISO can reduce ERS-induced injury by inhibiting Ca^2+^ overload, reducing the generation of reactive oxygen species (ROS), and decreasing apoptosis. These results proposed that ISO inhibited the TGF-β/Smad signaling pathway and relieved oxidative stress, thus inhibiting HSC activation, and preventing liver fibrosis (Yang et al., 2016). Therefore, ISO can be seen as an anti-fibrotic drug to prevent chronic liver disease.

### 5.3 Neuroprotection

Nerve apoptosis is a common feature of many neurodegenerative diseases such as Alzheimer’s disease, Parkinson’s disease, Huntington’s disease, and amyotrophic lateral sclerosis (Mattson, 2000). Protein kinase C epsilon (PKCƐ), members of the PKC family characterized as a calcium-independent and phorbol ester/diacylglycerol-sensitive serine/threonine kinase, is abundantly expressed in neurons and has been found that participates in various effects in neurons (Chen et al., 2021). Accumulating evidences have confirmed that PKCƐ signaling is involved in alleviating Ca^2+^ depletion and ROS production in ER and inhibiting apoptosis (Rieusset et al., 2016). ISO, functioning as a ROS scavenger, effectively inhibits apoptosis. Furthermore, ISO demonstrates the ability to protect ERS-induced apoptosis in N2a cells, and the protective effects are involved in PKCƐ-mediated Ca^2+^ homeostasis and inhibition of ROS. The GRP78 protein is recognized as a multifunctional protein that plays a pivotal role in the endogenous mechanisms of neuroprotection (Casas, 2017). The effects of ISO against ERS injury in N2a cells were detected by cell viability, the levels of Ca^2+^, apoptosis, and ROS. Results demonstrated that ISO could elicit protective effects against ERS injury in N2a cells through depending on GRP78 (Qiu et al., 2017).

### 5.4 Kidney protection

Renal injury is closely associated with diabetes, particularly in the context of diabetic kidney disease (DKD), a devastating microvascular complication associated with diabetes mellitus. Recently, the major focus of glomerular lesions of DKD has partly shifted to diabetic tubulopathy as a significant aspect of glomerular lesions in DKD, given its close correlation with renal insufficiency and patient prognosis tied to tubular atrophy and interstitial fibrosis. The prevalent form of diabetes, often identified as type 2 diabetes, is on the rise globally. Although surgery can ameliorate the condition, postoperative glycemic control remains an unsolved issue (Curran and Kopp, 2022), leading to serious complications such as diabetic nephropathy, atherosclerosis, retinopathy, along with oxidative damage to tissues and cells (Kalai et al., 2022). The type 2 diabetic rat model was widely used by a high-fat diet plus streptozocin injection. In an animal model study, rats with the established type 2 diabetic model were subsequently subjected to ISO treatment, and their blood glucose levels were assessed. To evaluate renal function, measurements were taken for urinary osteopontin, kidney injury molecule-1 (KIM-1), and albumin. The assessment of renal NF-κB signaling activity involved measurements of NF-κB p65, phosphor-NF-κB p65, inhibitor of NF-κB (IκBα) and phosphor-IκBα, and the NF-κB p65 DNA-binding activity. The investigation of downstream inflammatory mediator TNF-α, interleukin-1β (IL-1β), IL-6, intercellular adhesion molecule-1 (ICAM-1) and TGF-β1 of the NF-κB signaling pathway were investigated to evaluate the renal inflammatory response. Research results showed ISO had renoprotective effects in type 2 diabetic rats, and the recovery of renal damage may be associated with the inhibition of the NF-κB signaling pathway.

## 6 Other *in vitro* pharmacological potentials of ISO

In this ongoing investigation, in addition to elucidating the *in vitro* antitumor and organ protection properties of ISO, we comprehensively review additional *in vitro* pharmacological potentials of ISO.

### 6.1 Anti-inflammatory effects

The consensus holds that various injuries can trigger inflammation within the body. Throughout this process, a multitude of inflammatory cells, pro-inflammatory factors, chemokines, and related mechanisms mediate the progression of inflammation. Given the pervasive role of inflammation in numerous disease processes, the exploration and development of new anti-inflammatory drugs offer promising prospects for patients and clinical applications. NF-κB is known to be an important transcription factor that regulates a wide range of inflammatory genes (Zhou et al., 2022). Studies have shown that the anti-inflammatory effect of ISO is achieved by blocking the JNK and AKT/IKK signaling pathways, relying on the target gene of NF-κB, which may be one of the pharmacological mechanisms of ISO action in the treatment of acute inflammatory diseases (Yang et al., 2013). In addition, TNF-α activates human bronchial epithelial cells BEAS-2B to an inflammatory state, and ISO acts on this cell to attenuate bronchitis. Research also showed that ISO could inhibit TNF-α-induced inflammation, proliferation, and migration by regulating MAPK and NF-κB signaling pathways. These findings underscore ISO’s therapeutic role in asthma, specifically in improving TNF-α-induced airway inflammation and airway remodeling (Ren et al., 2021).

### 6.2 Anti-oxidant effects

Flavonoids exhibit various biological effects, including antioxidant effects (Seo et al., 2014). In a study investigating the effects of ISO on human retinal pigment epithelial cells (RPE), the results demonstrated that pretreatment of RPE cells with ISO significantly protected cell viability against oxidative stress. In addition, ISO pretreatment inhibited hydrogen peroxide (H_2_O_2_) induced ROS production and Caspase-3 activation in RPE cells. Furthermore, ISO pretreatment significantly increased the phosphorylation of PI3K and AKT in RPE cells exposed to H_2_O_2_, compared with cells treated with H_2_O_2_ alone. Collectively, these findings demonstrate that ISO protects RPE cells from oxidative stress-induced cell death, and this effect was associated with the activation of the PI3K/AKT signaling pathway. Therefore, ISO may be considered a potential antioxidant, useful for the prevention of age-related retinal macular degeneration.

### 6.3 Anti-viral effects

The global incidence of Coronavirus Disease 2019 (COVID-19) cases and related fatalities continues to increase. While the 2023 Nobel Prize in Physiology or Medicine acknowledged the pioneering contributions of mRNA vaccine technology in combating the coronavirus pandemic, the quest for more effective treatments for severe inflammation and acute lung injury resulting from new coronavirus infection remains urgent (Patel et al., 2021). Studies have reported that angiotensin-converting enzyme 2 (ACE2) has been identified as an infection receptor for severe acute respiratory syndrome (SARS-CoV-2), thus finding drugs that target and inhibit its expression will be the key to treating coronavirus (Barlow et al., 2020). In one previously published study, it was observed that ISO show strong retention to ACE2 overexpression in human embryo kidney HEK293 cells. ISO could interact with ACE2, the functional receptor for SARS-CoV-2, thus preventing SARS-CoV-2 spike pseudotypes viral entry and infection of human cells expression ACE2, which suggested that ISO might be an ACE2-spike protein interaction blocker. This suggests that ISO may serve as an ACE2-spike protein interaction blocker, positioning it as a potential therapeutic candidate against COVID-19 (Zhan et al., 2021).

### 6.4 Anti-coagulant effects

There is evidence of the effectiveness of a healthy diet and lifestyle in preventing cardiovascular disease (CVD) (Casas et al., 2018). Numerous investigations indicate that t flavonoids, such as ISO and its derivatives present in fruits and vegetables, can reduce the risk of CVD (Eccleston et al., 2002; Skalski et al., 2019a; Skalski et al., 2019b). Platelet activation is a causal factor in cardiovascular disorders, playing a fundamental role in thrombus formation, atherogenesis, and the progression of atherosclerotic lesions (Olas, 2017). Inhibiting platelet activation through natural products is considered as a central target to prevent thrombus formation (Méndez et al., 2020; Kamran et al., 2021). Studies have shown that ISO helps reduce platelet activation, thereby reducing the risk of thrombosis and cardiovascular disease. Additionally, the link between the development of cardiovascular disease and mitochondrial damage is well known. ISO also has antiplatelet activity by inhibiting mitochondrial bioenergetics. ISO may be a promising scaffold compound to develop new antiplatelet agents with specific action on thrombotic diseases without obvious cytotoxic effects.

The evidence suggests that this flavonoid may play an important role in health maintenance and potentially protect against cardiovascular disease by inhibiting platelet function, thereby reducing the risk of thrombosis.

### 6.5 Anti-osteoporosis effects

Osteoporosis, characterized by a decrease in bone mass, is widely recognized as a major public health problem. Osteoporosis is a chronic epidemic that can lead to increased bone fragility, thereby elevating the risk of fractures Various flavonoids had been reported to hold potent inhibitory effects on osteoclastic bone resorption rather than a stimulatory effect on bone formation *in vitro*, including ISO (Yang et al., 2013). Cultured with a bone-resorbing factor parathyroid hormone (PTH), ISO caused a significant increase in osteoclast-like cell formation in mouse marrow culture *in vitro*, and the osteoclastogenesis was markedly suppressed in the presence of ISO. These results provide insights into the pharmacological mechanisms of ISO in treating osteoporosis.

### 6.6 Anti-adipogenicity effects

Adipose tissue is an endocrine-type tissue that plays a central role in regulating energy homeostasis. Excessive fat accumulation resulting in obesity can induce mitochondrial dysfunction in fat cells, disrupting homeostasis and leading to lipodystrophy syndrome, accompanied by metabolic and cardiovascular complications (Vernochet et al., 2014). Mitochondrial dysfunction in various tissues has been linked to the development of obesity and type-2 diabetes. The peroxisome proliferator-activated receptor γ coactivator-1α (PGC-1α) serves as the metabolic regulator of mitochondrial biogenesis (Puigserver and Spiegelman, 2003). PGC-1α regulates mitochondrial biogenesis and function through induction of the expression of nuclear respiratory factors (NRFs), NRF-1 and NRF-2 (Chen et al., 2021). Additionally, PGC-1α indirectly regulates the expression of mitochondria DNA (mDNA) transcription by increasing the expression of mitochondrial transcription factor A (TFAM) (Kelly and Scarpulla, 2004). In a study involving 3T3-L1 adipocyte, ISO treatment stimulated the differentiation mRNA levels of mitochondrial genes, including PGC-1α, NRFs, TFAM, and carnitine palmitoyl transferase-1α (CPT-1α) (Lee et al., 2018). Specifically, lipid and triglyceride accumulated intracellularly, and glycerol-3-phosphate dehydrogenase (GPDH) activity decreased in ISO-treated cells. The mRNA levels of adipogenic genes, such as the proliferator-activated receptor-γ (PPAR-γ), and adipocyte protein 2 (aP2), were all inhibited by ISO. Therefore, ISO may be useful as a potential food ingredient to prevent obesity-associated mitochondrial dysfunction.

### 6.7 Anti-apoptosis effects

Apoptosis, a form of programmed cell death initiated by endogenous and external death signals, involves the expression of apoptosis-related proteins—a principal mechanism of various anti-tumor drugs, including ISO. A study investigated ISO’s potential to inhibit oxidized low-density lipoprotein (ox-LDL) induced cell apoptosis in THP-1-derived macrophages. Ox-LDL induces apoptosis in macrophages via oxidative stress injuries by promoting the high intracellular expression of ROS and NOX activity plays a critical role in ROS production (Dong et al., 2022). ISO was observed to inhibit ox-LDL-induced ROS production and NOX activity in macrophages, indicating its anti-oxidative activity of ISO. ISO showed significant inhibitory effects on ox-LDL-induced THP-1-derived macrophage injuries via decreasing ROS levels, lipid deposition, and caspase-3 activation, restoring mitochondrial membrane potential, reducing the number of terminal deoxynucleotidyl transferase-mediated dUTP nick end-labeling (TUNEL) positive cells, and ultimately regulating apoptosis-related proteins. These results unequivocally establish ISO’s role in reducing endothelial cell apoptosis.

### 6.8 Immunomodulation effects

Pulmonary fibrosis is a chronic and progressive disease characterized by alveolar epithelial injury and abnormal collagen production (King et al., 2011; Wolters et al., 2014). Patients with pulmonary fibrosis often exhibit those symptoms, thus pulmonary function is irreversibly lost (Raghu et al., 2011). Safer drugs with improved efficacy are needed for the treatment of pulmonary fibrosis. Pulmonary fibrosis is caused by the abnormal proliferation of myofibroblasts and fibroblasts, which secrete excessive extra-cellular matrix (ECM) proteins (Raghu et al., 2015). EMT is a process in which polarized immotile epithelial cells are converted to motile mesenchymal cells. An approach targeting EMT might be promising in the treatment of pulmonary fibrosis (Takemasa et al., 2012). There were studies revealed that ISO inhibited EMT in kurarinone in bleomycin (BLM)-induced pulmonary fibrosis via the PERK pathway. Those findings provide novel insights into the pharmacological potential of ISO in pulmonary fibrosis therapeutics.

**FIGURE 5:**
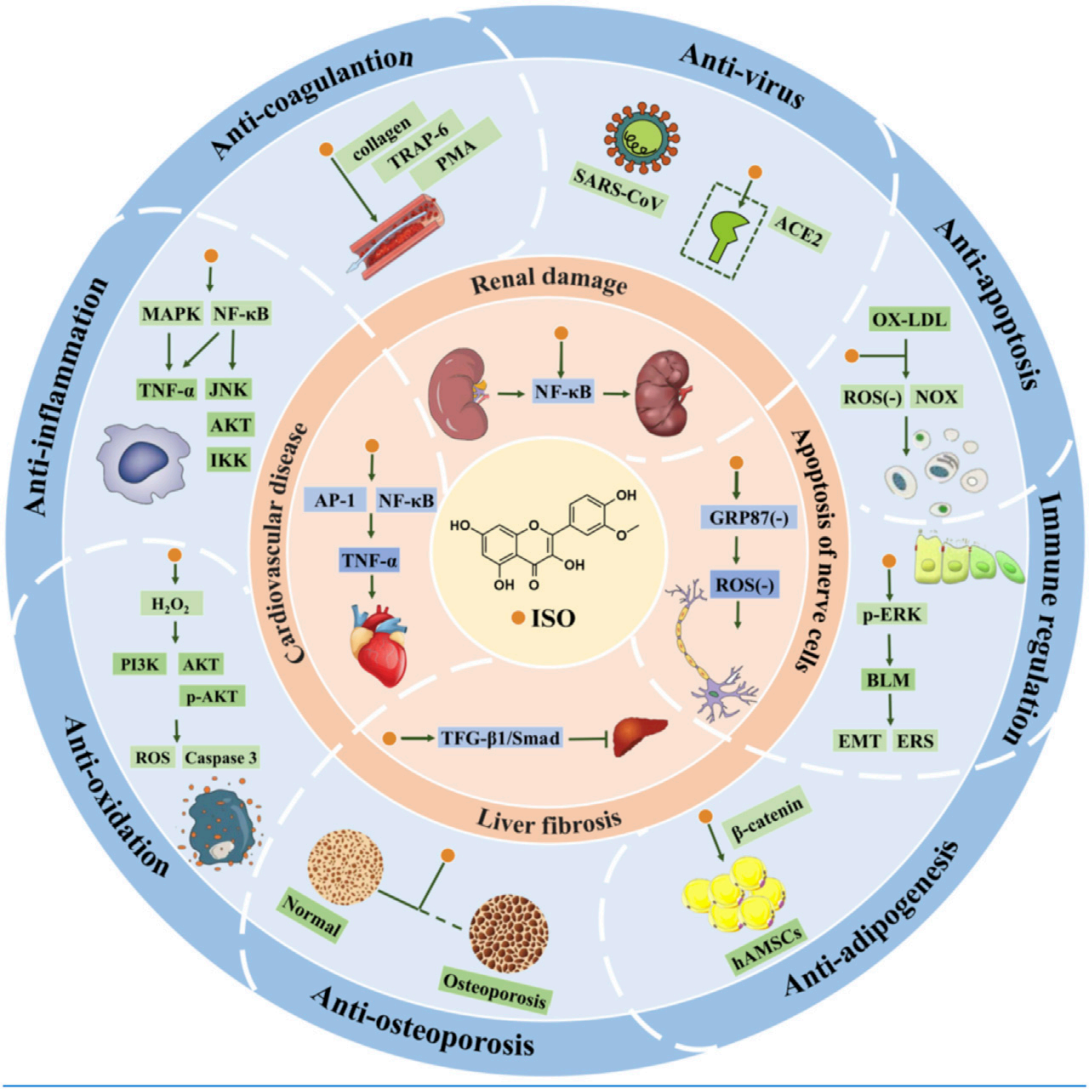
Organ protection and other pharmacological activities *in vitro* of ISO.

In summary, Figure 5 schematically depicts the other *in vitro* pharmacological properties and underlying mechanisms of ISO.

## 7 Conclusion and future perspectives

Natural small molecule compounds, including ISO, owing to their low toxicity and ready availability, have garnered attention as a focal point in recent disease research (Kim et al., 2022; Jalil et al., 2023). Cancer is a serious public health problem that imposes considerable burden on healthcare systems worldwide. Due to its inherent heterogeneity influenced by multiple factors, the complex and diverse pathology of cancer presents challenges across prevention, diagnosis, treatment, and survival. Despite advancements in cancer treatment, achieving a complete cure remains elusive. Besides cancer, some other diseases such as traumatic brain injury, ischemic stroke, myocardial ischemia-reperfusion injury, acute lung injury, acute kidney injury, etc., also severely threaten human health. ISO belongs to one of the main compounds of flavonoids, predominantly extracted from *H. rhamnoides L.* (Kalai et al., 2022), exhibits noteworthy anti-cancer effects *in vitro*. Nevertheless, while persuasive studies have affirmed ISO’s anti-cancer effects, the specific mechanism remains incompletely understood. Current research on ISO possesses limitations, including a restricted scope and insufficient depth. The precise regulatory role of ISO in tumor-related genes or related metabolic pathways remains unclear. Future exploration should encompass toxicological and pharmacokinetic effects of ISO, emphasizing combination approaches with other drugs to achieve the ideal anti-cancer purpose. Further elucidation of ISO’s specific role, targets, and mechanisms in anti-cancer applications is imperative.

Beyond its anti-cancer activity *in vitro*, this review has outlined additional pharmacological potentials of ISO, including anti-inflammatory, antioxidant, and anti-apoptosis effects. The traditional sources, phytochemistry, and pharmacology activities of ISO, rooted in ancient classics and modern research, offer a comprehensive perspective for future exploration.

Significantly, the primary source of ISO, the fruit of *H. rhamnoides L.*, exerts dual functions as medicine and food, enriched with over 190 identified compounds. ISO and its derivatives represent pivotal active ingredients, showcasing its prominence in *H. rhamnoides L*. It is expected that more compounds of these categories will unveil additional compounds within these categories. Moreover, research has shown that both crude extracts and active components of *H. rhamnoides L*. possess a diverse array of pharmacological activities. These activities span anti-inflammation, anti-tumor, anti-oxidation, antiviral and organ protective effects, cardiovascular protective effects, neuroprotective function, hepatoprotective activity, and more. These modern pharmacological studies supported most traditional uses of *H. rhamnoides L*. fruit as folk medicine. *Hippophae rhamnoides L.,* is widely used in medicine by the Chinese Pharmacopoeia and the World Pharmacopoeia (Geng et al., 2022). Notably, among the constituents, ISO accounts for the most important and representative ingredients of *H. rhamnoides L*. It is rich in nutrients, bioactive substances and various amino acids required by the human body, especially high in vitamins. In addition, it has the effect of strengthening physical fitness and improving immunity (Ciesarová et al., 2020). Nevertheless, there remain gaps in systematic research on *H. rhamnoides L*., necessitating urgent exploration of molecules both upstream and downstream of ISO due to the current lack of in-depth evidence. Furthermore, the pharmacological activities of ISO should undergo thorough confirmation through additional animal models experiments utilizing diverse animal models and combined with clinical applications (Surendran et al., 2021). In conclusion, the fruit of *H. rhamnoides L.* is an important edible medicinal herb with extensive pharmacological activities and great value in both medicine and food. Extracted from the fruit, ISO presents an ideal medicinal profile. However, a more in-depth and comprehensive studies on clinical utility is imperative to ascertain the safety and availability of ISO (Willcox et al., 2024). Due to the complexity of the existing small components in natural products and the unclear mechanism of action, there are challenges such as difficulty in extraction and processing, as well as poor bioavailability, pose hurdles for their clinical use in treatment. Therefore, it is important to recognize the safety and potential side effects of ISO with a high degree of product specificity, and further research is needed to better understand their optimal dose, long-term effects, and potential therapeutic uses for *in vivo* adverse reactions. Further investigations may focus on: 1) persistent exploration of toxicological and pharmacokinetic effects of ISO; 2) evaluating potential suboptimal potency in the clinical application of therapeutics targeting ISO; 3) probing into other derivatives of ISO and their roles; and 4) continuing to study other molecules upstream and downstream of ISO, which may be potential therapeutic targets in the future. Despite the discovery of multiple compounds in *H. rhamnoides L.*, ISO exerts the most frequently reported pharmacological potentials. Although the concept of ISO as a “Medicine food homology” agent has achieved solid foundations, current efforts are insufficient. Polyphenolic compounds refer to the general term of plant components with several phenolic hydroxyl groups in their molecular structure, including flavonoids, tannins, phenolic acids and anthocyanins (Vissenaekens et al., 2022). How to choose a suitable positive or negative control compound to determine a specific monomer polyphenol compound and avoid some interfering polyphenols substances which may result in false positives is also a core problem to be resolved for future researchers in this field (Xia et al., 2023). Lastly, the precise molecular mechanisms of ISO in some other diseases remains a subject worthy of further study. Consequently, systematic studies on the phytochemistry and bioactivities of ISO will undoubtedly be the key direction of future research. This comprehensive review aims to illuminate and guide the development and application of ISO in the pursuit of scientific advancements.
